# PGLYRP4 Enhances *Shigella flexneri* Virulence by Promoting *virF* Transcription via the CpxA/R Two‐Component System

**DOI:** 10.1002/mbo3.70156

**Published:** 2025-11-09

**Authors:** Rita Trirocco, Gianni Prosseda

**Affiliations:** ^1^ Institute Pasteur Italia, Department of Biology and Biotechnologies “Charles Darwin” Sapienza University of Rome Rome Italy

**Keywords:** host‐pathogen interaction, innate immunity, PGRP, *Shigella*, virulence regulation

## Abstract

The human gastrointestinal tract provides a complex environment for bacterial pathogens, necessitating their adaptation to host defenses and microbiota. *Shigella*, a Gram‐negative bacterium responsible for bacillary dysentery, has evolved sophisticated mechanisms to regulate its virulence in response to intestinal signals. This study focuses on the role of peptidoglycan recognition protein 4 (PGLYRP4), a component of the host's innate immune system, in modulating *Shigella*'s virulence. We demonstrate that PGLYRP4, at sub‐bactericidal concentrations, significantly induces the transcription of the virulence regulator *virF*, through the CpxA/R TCS activation, therefore enhancing *Shigella*'s infectivity without compromising bacterial viability. Moreover, our findings suggest that *Shigella* has developed an increased capacity to respond to oxidative stress, including that induced by PGLYRP4, through the basal upregulation of genes involved in detoxifying reactive oxygen species. This adaptation likely helps the pathogen counteract the bactericidal effects of PGLYRP4. Based on the results of our experiments and the literature, we hypothesize that *Shigella* uses PGLYRP4, which is produced by stimulated enterocytes in response to cytokines released by pyroptotic macrophages, as a molecular cue to enhance its ability to invade enterocytes. This study contributes to improving our understanding of bacterial pathogens' adaptation strategies by showing that they can evolve to compete more effectively with their hosts by using factors of the hosts' arsenal.

## Graphical abstract

- *Shigella* can use the host's innate immune system to optimize the expression of its virulence factors.
- The PGLYRP4 protein, a member of the host's innate immune system, increases VirF expression in *Shigella* by stimulating the CpxA/R TCS, thereby enhancing the expression of the virulence system.
- PGLYRP4 ultimately improves *Shigella* invasiveness in host cells.

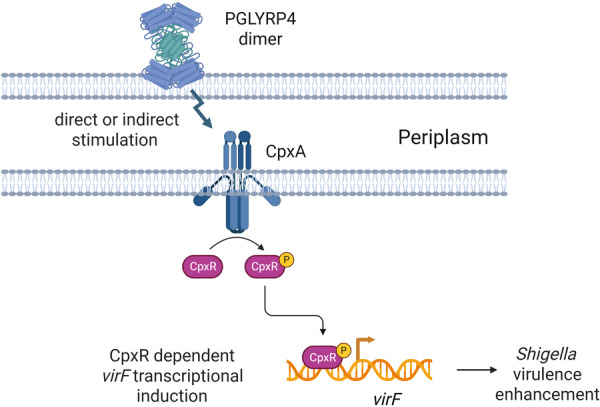

## 1 Introduction

The human body provides a multitude of niches for bacterial pathogens to colonize. These pathogens have evolved characteristics and mechanisms to survive and adapt to the specific environment they encounter during the infection. This is especially evident in the human gastrointestinal tract, where bacterial pathogens must withstand the presence of toxic substances, such as hydrochloric acid and bile salts, cope with the “colonization resistance” by the intestinal microbiota, and evade the host's immune defenses to thrive. Highly adapted human pathogens have evolved the capacity to finely regulate the expression of virulence systems at appropriate times and circumstances by sensing the intestinal compounds originating from the microbiota or host tissues, and even the host immune‐specific signals and factors (Hitzler et al. 2025). *Shigella* can rightfully be counted among these microorganisms, as its pathogenic evolution is estimated to have begun with the emergence of *Homo sapiens* about 35,000–270,000 years ago (Pupo et al. 2000; The et al. 2016). This Gram‐negative bacterium, closely related to *Escherichia coli*, is the etiological agent of human bacillary dysentery, responsible for around 165 million yearly cases worldwide, with 600,000 fatal outcomes, mainly among children in Africa and South Asia (The et al. 2016). The members of the genus *Shigella (S. dysenteriae, S. flexneri, S. boydii,* and *S. sonnei)* infect hosts via the fecal‐oral route with a very low bacterial load (10‐100 bacterial cells) (Killackey et al. 2016).

Once *Shigella* reaches the human colon, it invades the intestinal mucosa, causing severe inflammation and diarrhea. This process involves *Shigella* crossing the mucosa through M‐cells, evading macrophages via pyroptosis, invading enterocytes, and eventually spreading to adjacent cells (Mattock and Blocker 2017; Pasqua et al. 2017). The genetic determinants of *Shigella* pathogenicity are located on the pINV virulence plasmid and are regulated by three transcriptional regulators, VirF, VirB, and MxiE (Di Martino, Falconi, et al. 2016; Haidar‐Ahmad et al. 2023). VirF activates the *icsA* and *virB* genes (Tran et al. 2011), the former encoding the protein responsible for the intracellular movement of *Shigella*, while the product of the latter induces the transcription of invasion‐related genes, including *mxiE*, whose product controls the factors required for the intracellular infection phase (Beloin et al. 2002; Mavris et al. 2002). *Shigella* has also evolved the ability to control the expression of its virulence mechanisms at the right time and place by detecting compounds in the gut. Some of these adaptive mechanisms are well‐characterized. At human body temperature, the *virF* promoter DNA structure undergoes a temperature‐dependent transition that activates *virF* transcription and virulence system expression (Falconi et al. 1998; Prosseda et al. 2004). Bile salts regulate *Shigella* virulence in the intestine by promoting type III secretion system (TTSS) maturation and cell adhesion as well as modulating biofilm formation (Faherty et al. 2012; M. Kim et al. 2009; Nickerson et al. 2017). In the colon, leucine increases virulence gene expression through SsrV small RNA‐mediated induction of LrhA, a transcriptional activator of *virF* (Li et al. 2024). Some fatty acids suppress virulence in the small intestine by inhibiting VirF protein activity (Trirocco, Pasqua, Tramonti, et al. 2023). Therefore, the reduction of such FAs in the colon or near the intestinal mucosa allows reactivation of VirF (Trirocco, Pasqua, Tramonti, Colonna, et al. 2023). In addition, increased oxygenation near intestinal capillaries further enhances *Shigella* virulence (Marteyn et al. 2012). Specific chemical signals, including pH, K^+^, and Na^+^ ions, are known to induce the expression of multiple efflux pumps, which facilitate the adaptation of *Shigella* within the host cells, including in harsh subcellular environments, such as the phagosomes in the intra‐macrophagic infection step (Coluccia et al. 2023; Pasqua et al. 2021, 2019). Further important evidence of *Shigella*'s ability to sense signals from the human gut and trigger specific responses comes from the involvement of two‐component systems (TCSs) in regulating the virulence of this pathogen and contributing to its survival, invasiveness, and ability to cause infection (Pasqua et al. 2022). TCSs are conserved signaling systems that enable cells to detect and react to various external or internal stimuli (Stock et al. 2000). TCSs consist of a sensor histidine kinase (HK) and a response regulator (RR). Depending on the presence or absence of signals, the HK can phosphorylate or dephosphorylate the RR, changing its DNA‐binding affinities. This affects the regulation of metabolic functions and cellular reactions, thus allowing organisms to adjust to changing environments (Gao et al. 2019). To date, six different TCSs have been implicated in the regulation of *Shigella* virulence in response to the environmental conditions experienced by this microorganism in the host environment. These are ArcA/B, PhoQ/P, EvgS/A, EnvZ/OmpR (Pasqua et al. 2022), PhoR/B (Li et al. 2024), and CpxA/R (Nakayama and Watanabe 1998; Pasqua et al. 2022). Among them, the role of TCS CxpA/R is crucial, as it directly controls the transcription of the *virF* gene and plays a role in the posttranscriptional regulation of the VirB protein (Mitobe et al. 2005). CpxA/R, which responds to changes in pH, is thought to be involved in suppressing the *Shigella* virulence system at low pH in the gastric environment. Interestingly, CpxA/R can regulate more than 70 *E. coli* genes responsible for environmental adaptations (Zhao et al. 2022), probably by responding to protein misfolding caused by several other stress conditions, as osmolarity and the presence of copper, ethanol, detergents, and chelators (Hunke et al. 2012; Raivio 2014).

In recent years, CpxA/R has been reported to mediate or contribute to in vitro bactericidal or bacteriostatic activity against Gram‐negative bacteria treated with peptidoglycan recognition proteins (PGRPs). PGRPs are innate immunity proteins conserved from insects to mammals (Royet and Dziarski 2007; Yoshida et al. 1996). Mammals, including humans, have four PGRPs, designated PGLYRP from 1 to 4, which are secreted by cells as disulfide‐linked homo‐ and heterodimers (Royet et al. 2011) in various tissues and organs, including the human gut (Kashyap et al. 2014). PGLYRP3 and 4 are expressed and secreted selectively by columnar absorptive cells in the small and large intestine (Dziarski and Gupta 2010). The presence of PGLYRP proteins in the human gut, and the involvement of the TCS CpxA/R in mediating both the biological effect of PGLYRP proteins in bacteria and the transcriptional induction of the master regulator of the *Shigella* virulence system, *virF*, has led us to investigate, for the first time as far as we know, whether PGLYRPs, despite being recognized for their bactericidal properties, could act as a host‐derived signal that influences the expression of bacterial virulence genes in *Shigella*. The results we obtained demonstrate that, at sub‐bactericidal concentrations, PGLYRP4 can induce *virF* transcription through CpxA/R, thereby enhancing infectious efficiency. This suggests that PGLYRP4 may play a role as a molecular cue in *Shigella* infection process, contributing to the precise regulation of the *Shigella* virulence system based on environmental conditions imposed by tissue location and stage of infection.

## 2 Materials and Methods

### 2.1 Bacterial Strains and General Growth Conditions

Bacterial strains used in this study are listed in Table S1. Bacteria were grown aerobically in lysogeny broth (LB) medium (Sigma‐Aldrich) at 37°C. To monitor the effect of PGLYRP1, PGLYRP2 (Origene; 1 mg/mL each), PGLYRP3 (R&D System; 50 µg), and PGLYRP4 (Abcam; 200 µg) activity, overnight cultures of M90T *S. flexneri* were diluted 1:50 in the same media and grown up to OD_600_ 0.1. Each PGLYRP, dissolved in water, was added to a final concentration of 0.05. 0.1, 0.2, 1, and 2 µg/mL, and bacterial cultures were grown either until the indicated time point or until reaching an OD_600_ of 0.6. Antibiotics were used at the following concentrations: ampicillin 100 µg/mL; kanamycin 30 µg/mL; streptomycin 10 µg/mL.

### 2.2 General Procedures

Standard procedures for total DNA and plasmid purification, transformation, and gel electrophoresis were followed (Trirocco, Pasqua, Tramonti, Colonna, et al. 2023; Wood 1983). Oligonucleotide sequences based on the M90T genome (CM001474.1) are listed in Table S2. PCR was performed with DreamTaq DNA polymerase (Thermo Fisher Scientific) or Ex Taq DNA polymerase (Takara) when higher fidelity was needed. *cpxA* or *cpxR* defective mutant M90T strains were constructed via one‐step gene displacement (Datsenko and Wanner 2000). The kanamycin resistance gene, amplified using pKD4 as template and *ΔcpxA_F*/*ΔcpxA _R* or *ΔcpxR_F*/*ΔcpxR_R* (Table S2), was electroporated into M90T, M90T *virF*‐FT, or M90T *virB*‐FT strains with the pKD46 plasmid expressing λ Red recombinase. Selected Km‐resistant clones were verified by colony PCR, using the *ΔcpxA_scrF*/*ΔcpxA_scrR* or *ΔcpxR_scrF*/*ΔcpxR_scrR* oligo pairs, and gel electrophoresis. The Km resistance cassette was removed from *cpxR* mutant strains using Flp/FRT recombination with Flipase from the pCP20 plasmid (Datsenko and Wanner 2000).

### 2.3 RNA Isolation and Quantitative Reverse Transcription Real‐Time PCR (qRT‐PCR) or qPCR

Bacterial RNA purification was carried out using the hot phenol extraction method (Livak and Schmittgen 2001). qRT‐PCR analysis was performed on a Real Time Step One Plus (Applied Biosystem) following the methodology previously described (Trirocco, Pasqua, Tramonti, et al. 2023). The reaction volume was 20 μL, containing 25 ng cDNA and Power SYBR Green PCR Master Mix (Applied Biosystem). The *nusA* gene transcript was used as a reference. For each point, a technical triplicate was performed. The amounts of the transcripts were analyzed using the 2^−ΔΔCt^ method (Fanelli et al. 2020), and the results were indicated as n‐fold increase relative to the reference sample. A similar approach was used to compare the amount of *gfp* genes, relative to *nusA* genes, in the *Shigella* M90T strain and *E. coli* MG1655 strain. The difference was that genomic DNA was used as a template instead of cDNA (qPCR). All the primer pairs used for qRT‐PCR and qPCR, listed in Table S2, were designed with Primer Express software v2.0 (Applied Biosystem).

### 2.4 SDS‐PAGE and Immunoblot Analysis

Total protein extracts from PGLYRPs‐treated and untreated bacteria were run in a 12.5% SDS‐PAGE, then proteins were transferred to nitrocellulose membranes (Hybond‐P, Millipore) and analyzed by immunoblotting. A protein molecular weight marker (Thermo Fisher Scientific) was included in each electrophoresis run to determine the proteins' molecular weight. Immunoblotting was carried out with monoclonal ANTI‐FLAG antibody (Sigma‐Aldrich), and anti‐mouse was used as the secondary antibody. Specific bands were visualized by enhanced luminol‐based chemiluminescent substrate (Thermo Fisher Scientific) and detected using the Chemidoc XRS imaging systems (Bio‐Rad). The Signal Accumulation Mode protocol was used, enabling us to select the optimal image with the best signal‐to‐noise ratio, avoiding bandwidth saturation. Densitometric analysis was performed using the Image Lab Software (Bio‐Rad). The OmpA protein was used as a reference.

### 2.5 Cell Cultures and Infections

Infection experiments using human epithelial Caco‐2 cells were performed as previously described (Trirocco, Pasqua, Tramonti, et al. 2023). Briefly, cells were cultured in DMEM with 10% FBS, L‐glutamine, and PS (DF10) at 37°C with 5% CO₂. Cells were seeded in six‐well plates at 4 × 10⁵ cells/well in DF10. After 48 h, cells were serum‐starved overnight in DF0.5. 2 h before infection, the medium was replaced with serum‐free DMEM with L‐glutamine. Infection occurred at an MOI of 100. Postinoculation, plates were centrifuged at 750 × g for 15 min and incubated for 45 min at 37°C in 5% CO₂. Extracellular bacteria were removed by washing the infected cells three times with PBS. Fresh DMEM containing gentamicin was then added, and the cells were incubated at 37°C for up to 4 h. At each time point (T1, T2, T3, and T4), corresponding to 1‐, 2‐, 3‐, and 4‐h postinfection, three wells were washed again with PBS to eliminate any remaining extracellular bacteria. The intracellular bacteria were then recovered by lysing the cells, resuspending the lysate in 0.9% NaCl, and plating serial dilutions on LB agar. Colony‐forming units (CFUs) were counted and expressed as CFU/mL. Time point T0 represented a control in which the infection was maintained in DMEM containing gentamicin for only 5 min before processing.

### 2.6 Plaque Assay

Plaque assay was performed as previously described (Coluccia et al. 2023). Caco‐2 cells (5 × 10⁶) were seeded in 60 mm dishes with DF10 medium. After reaching confluence in 24 h, cells were serum‐starved overnight in DF0.5. 2 h before infection, DMEM with 2 mM L‐glutamine was added. Infections at MOI 0.001 were followed by centrifugation at 750 × g for 15 min and incubation at 37°C with 5% CO₂ for 45 min. Extracellular bacteria were removed by washing with PBS. An agarose overlay with DMEM, gentamicin (100 μg/mL), 5% FBS, and 0.5% agarose was applied, and cells were incubated for 72 h at 37°C. Agarose overlay was removed, monolayers fixed with ethanol, and stained with Giemsa. Plaques were imaged with ZEOTM fluorescent cell imager (Bio‐Rad) and quantified using ImageJ software.

### 2.7 Growth Kinetics and Minimum Inhibitory Concentration (MIC) Assay

Bacterial growth kinetics, with or without 0.625 mM H_2_O_2_, were measured using a CLARIOstar plate reader (BMG LABTECH). The MIC was determined using the broth microdilution method in 96‐well microtiter plates. Serial two‐fold dilutions of the antimicrobial compound were prepared in LB. Bacterial cultures were added at a final OD_600_ 0.1. Plates were incubated at 37°C for 8 h, and MICs were defined as the lowest concentration of the compound that completely inhibited bacterial growth, detected with the CLARIOstar plate reader (BMG LABTECH).

### 2.8 Oxidative Stress

Plasmid pLA40, a pFPV25 derivative carrying the *ahpC* promoter fused to *gfp* reporter, was used to assay the intracellular oxidative stress in MG1655 *E. coli* and M90T *S. flexneri* strains (Aussel et al. 2011). Bacteria were grown in M9 complete medium with or without 0.625 mM H_2_O_2_. A volume of 200 μL of each sample was transferred to a 96‐well microtiter plate. The bacterial density (abs at OD_600_) and fluorescence (405–510 nm, Ex/Em) were measured with CLARIOstar plate reader (BMG LABTECH). The reporter activity was determined as relative fluorescence units normalized to the OD_600_ of each sample. Background fluorescence was subtracted by assaying the corresponding MG1655 pFPV25 and M90T pFPV25 controls.

### 2.9 Statistical Analysis

The statistical analysis was performed using GraphPad Prism version 5.0 (GraphPad Software). Depending on the sets of data, either the student's t‐test or the two‐way analysis of variance was employed. All data are expressed as mean ± standard deviation (SD). A value of *p* < 0.05 was considered to be statistically significant.

## 3 Results

### 3.1 PGLYRP4 Induces the Expression of VirF and Does Not Affect the Viability of *Shigella*

**Figure 1:**
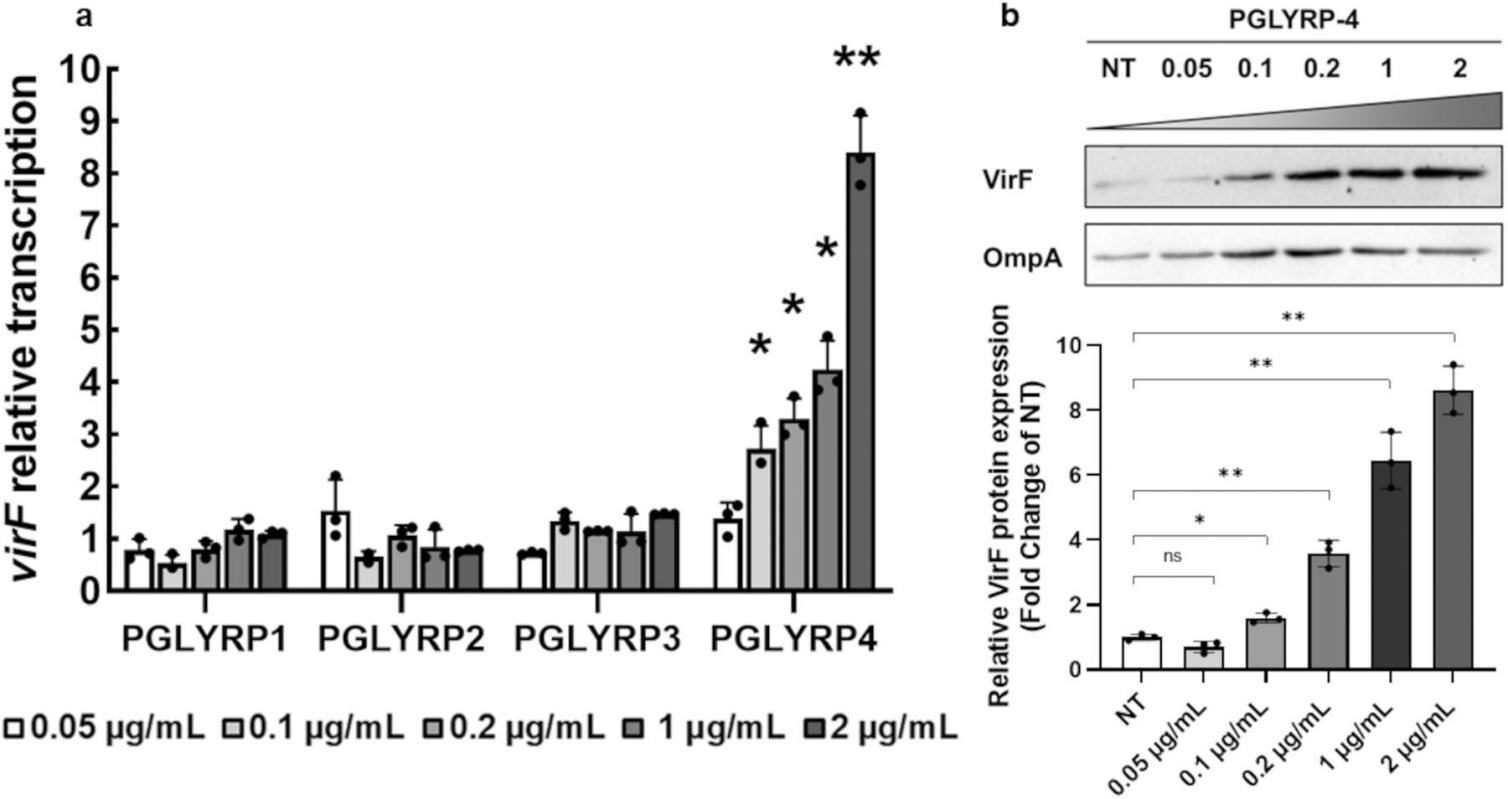
PGLYRP4 induces the expression of VirF in *S. flexneri*. The graph shows the relative transcription (a) and translation (b) levels of the *virF* gene and the VirF protein in *S. flexneri* strain M90T grown in the presence of increasing concentrations (0.05–2 µg/mL) of PGLYRP1‐4 proteins. (a) RT‐qPCR results are compared to the corresponding untreated sample set to 1 (not shown). Results are the average of at least three independent experiments performed in triplicate (n = 3). (b) Western blot images are representative of three independent experiments (top panels) (uncropped images in Figure S9), while the densitometric analyses of all experiments were used to calculate the values shown under the corresponding WB. Values were obtained by normalizing the VirF protein levels to those of the OmpA protein and are presented relative to the untreated samples (NT) set to 1. Error bars represent SD. Statistical significance was determined with a paired two‐tailed Student's t‐test using the measurements of the untreated and treated samples as a data set. *p ≤ 0.05; **p ≤ 0.01; ns, not significant.

To test whether PGLYRP proteins significantly influence *virF* expression, we analyzed *virF* transcription and translation in *S. flexneri* in the presence of purified PGLYRP proteins. Given the bacteriostatic and bactericidal properties of PGLYRP proteins, it was important to ensure that the concentrations used did not affect the growth of *Shigella*. To this end, we monitored the growth of the *S. flexneri* M90T strain in LB at 37°C in the presence of increasing concentrations of PGLYRP1, 2, 3, or 4 (0.05–2 µg/mL) (Figure S1). The growth curves show no significant differences, confirming that PGLYRP 1, 2, 3, and 4 have neither bacteriostatic nor bactericidal effects at the concentrations used in this study. Then, we assessed the levels of *virF* transcripts by qRT‐PCR. Total RNA used to synthesize the template cDNA was extracted from *S. flexneri* M90T grown in LB at 37°C in the presence of increasing concentrations (0.05–2 µg/mL) of PGLYRP1, 2, 3, or 4. The results, obtained with *virF*‐ and *nusA*‐specific primers (Trirocco, Pasqua, Tramonti, et al. 2023), demonstrate that the PGLYRP1, 2, and 3 proteins do not affect *virF* transcription, while PGLYRP4 induces a dose‐dependent increase, reaching an eightfold level at the highest concentration used (2 µg/mL) (Figure 1a). To test whether the *virF* transcriptional induction by PGLYRP4 corresponds to an increased VirF protein synthesis, we performed a Western blot assay using total protein extracts from an M90T VirF‐FT *Shigella* culture, grown with PGLYRP4 under the same conditions as the transcriptional assay. The M90T VirF‐FT strain expresses a VirF flag‐tagged protein that can be immunodetected with anti‐FT specific antibody (Merck) (Di Martino, Romilly, et al. 2016). The outcome of this analysis shows a PGLYRP4 dose‐dependent VirF protein expression profile that is fully consistent with the result of the transcriptional analysis (Figure 1b), thus confirming the increased VirF expression and ruling out influences of PGLYRP4 on possible post‐transcriptional regulatory mechanisms of VirF expression.

Moreover, we assessed the time‐dependent induction of *virF* transcription by PGLYRP4 in *Shigella* cultures. Specifically, we monitored *virF* expression via qRT‐PCR in cultures treated with 2 µg/mL of PGLYRP4 over 90 min. The results (Figure S2) show a rapid increase in *virF* transcript over time, up to 60 min, followed by a slowdown at 90 min. This suggests either a time‐dependent PGLYRP4 induction of *virF* or that sustained growth of *Shigella* in the presence of PGLYRP4 could be necessary to trigger *virF* expression, thereby activating the *Shigella* virulence system.

### 3.2 PGLYRP4 Increases *S. flexneri* M90T Invasion of Epithelial Cells

**Figure 2:**
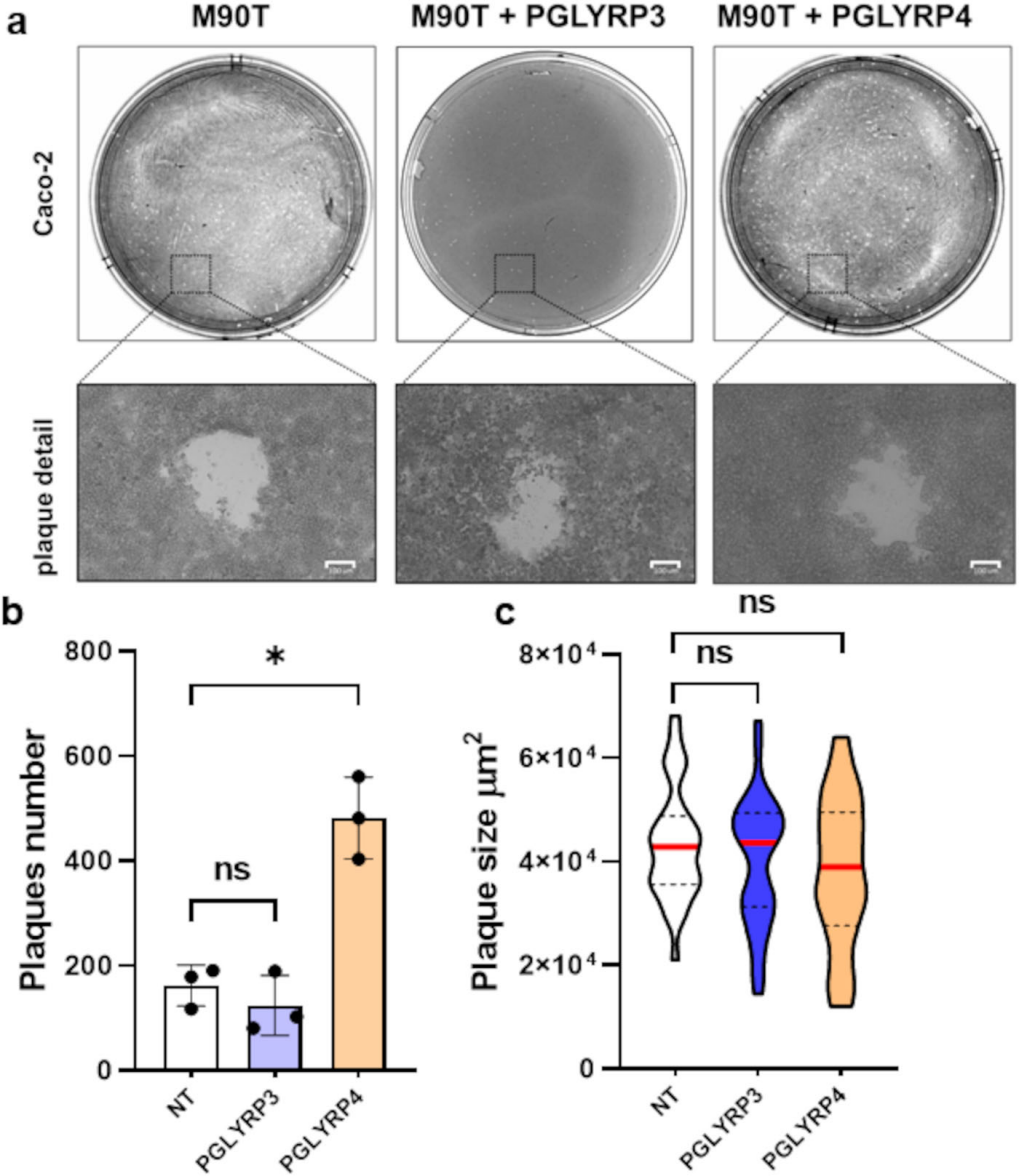
PGLYRP4 protein enhances *S. flexneri* invasion into epithelial cells but does not affect its spread. (a) Images of the plaque assay performed on a monolayer of Caco‐2 cells with *S. flexneri* M90T strain treated with no or 2 µg/mL PGLYRP3 or 4. Enlargements show representative plaques, showing that the plaque sizes are comparable. (b) The graph shows the mean number of plaques from three independent experiments (*n* = 3) and (c) the distribution of plaque size for each set of plaques (*n* = 40). Error bars (b) and dotted lines (c) represent the SD, while the average values are indicated by a red line. Statistical significance was determined with a paired two‐tailed Student's *t*‐test using the measurements of the untreated and treated samples as a data set. *p ≤ 0.01; ns, not significant.

**Figure 3:**
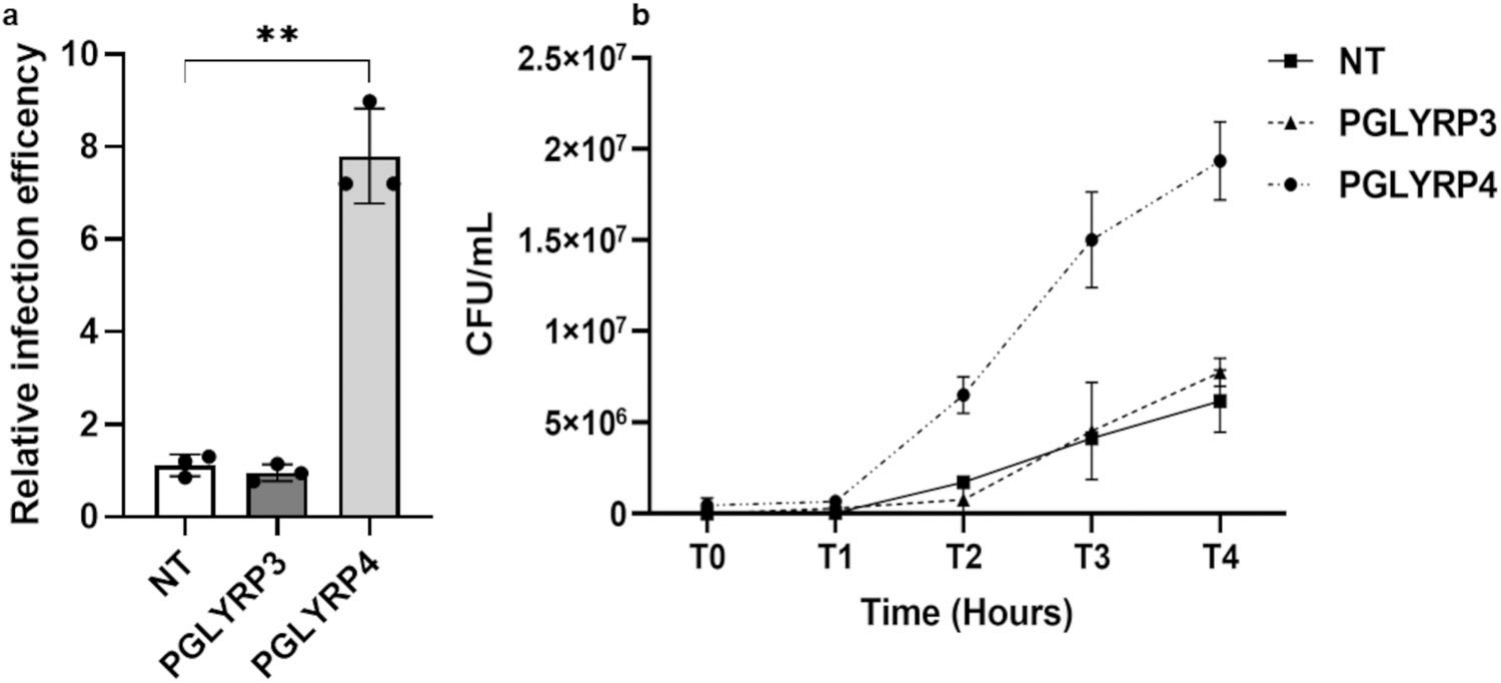
PGLYRP4 protein enhances *S. flexneri* invasion and proliferation in epithelial cells. Viable counts (CFU/mL) of intracellular bacteria were performed at T0 (a) to assess relative infection efficiency and over 4 h of infection (0–4) (b) to assess bacterial intracellular proliferation. Infections of Caco‐2 epithelial cells were performed at an MOI of 100 with *S. flexneri* M90T grown in the absence (NT) or with 2 µg/mL PGLYRP3 or 4. Results are averaged from at least three independent experiments (*n* = 3). Statistical significance was determined by a paired two‐tailed Student's *t* test (a) and the two‐way analysis of variance (b) (ANOVA; *p* = 0.0026). Error bars represent the SD.

VirF is the master regulator of *Shigella* virulence and net of any posttranslational regulatory mechanisms (Trirocco, Pasqua, Tramonti, Colonna, et al. 2023; Trirocco, Pasqua, Tramonti, et al. 2023), its increased transcription leads to the activation of the virulence system and thus the ability of *Shigella* to invade host cells. In light of this and considering the significant increase in VirF expression induced by the PGLYRP4 protein, we performed a plaque assay analysis to test whether PGLYRP4 could enhance the ability of *Shigella* to invade host cells and spread within an epithelium (Figure 2a). The number of plaques observed in the monolayer of Caco‐2 cells infected with *S. flexneri* M90T pretreated with 2 µg/mL of PGLYRP4 shows a 3.6‐fold increase compared with the untreated control (Figure 2b). Furthermore, it is interesting to note that the size of the plaques is comparable (Figure 2c), suggesting that the PGLYRP4‐mediated enhancement of the virulence system is no longer active intracellularly. We performed the same experiment with 2 µg/mL PGLYRP3, which does not induce *virF* transcription (Figure 1a). In this case, the number of plaques obtained with the pretreated sample was comparable to that of the untreated sample (Figure 2b), demonstrating that the PGLYRP4‐mediated increase in *virF* transcription is responsible for the enhanced invasive ability of *Shigella*. To further confirm the role of PGLYRP4 in improving *Shigella*'s ability to invade host cells and to assess its effect on intracellular proliferation, we carried out a gentamicin protection assay on Caco‐2 cells using the M90T *Shigella* strain grown in the absence or the presence (2 μg/mL) of PGLYRP4, and PGLYRP3 was used as a negative control. Infections were allowed to proceed for 4 h, and the number of intracellular bacteria was determined by viable cell counting on epithelial cell lysates. As shown in Figure 3a, the number of PGLYRP4‐treated M90T cells recovered at T0 is approximately seven times higher than that of untreated cells and of PGLYRP3‐treated controls, confirming the results obtained with the plaque assay. As for proliferation, PGLYRP4‐treated M90T cells exhibit a higher growth rate during the 4‐h infection period (T0–T4) than untreated and PGLYRP3‐treated cells (Figure 3b), probably due to the higher initial intracellular bacterial load caused by the improved invasive capacity (Figure 3a).

### 3.3 CpxA/R Is Involved in the PGLYRP4‐Mediated Virulence Regulation of *Shigella*

**Figure 4:**
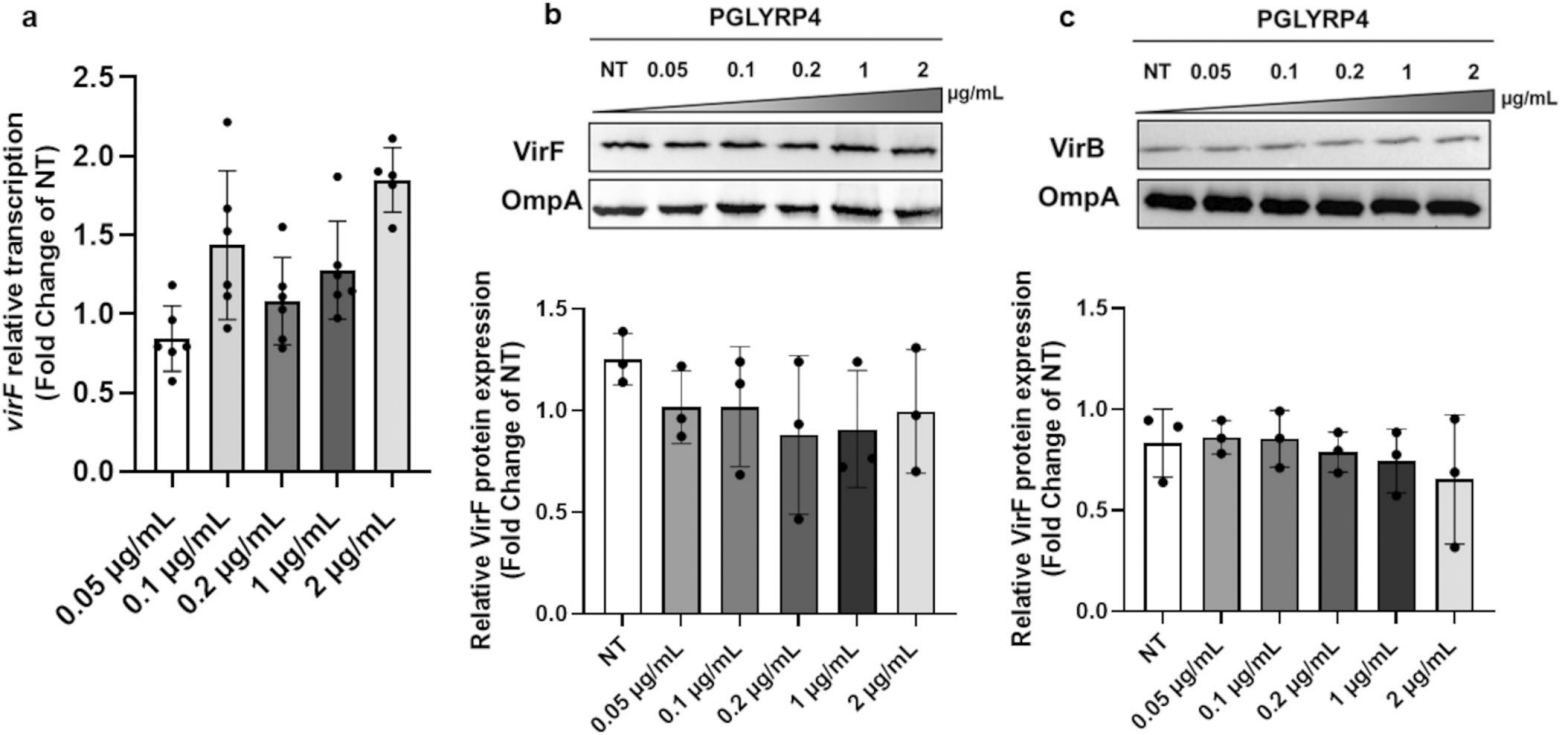
CpxA mediates the PGLYRP4 enhancement of the *S. flexneri* virulence system. The graphs show relative *virF* transcription (a) and VirF (b) or VirB (c) protein levels in *S. flexneri* M90T *cpxA* defective strain grown in the presence of increasing concentrations (0.05–2 µg/mL) of PGLYRP4 protein. Western blot images are representative of three independent experiments (top panels), while densitometric analyses of all experiments are shown under the corresponding representative WB (uncropped images in Figure 9S). Values were obtained by normalizing the VirF or VirB protein levels to those of the OmpA protein and are presented relative to the untreated samples (NT) set to 1. Error bars represent SD calculated with *n* = 6 for the transcriptional analysis (a) and *n* = 3 for the translational analysis (b and c). The lack of statistical significance was confirmed with a paired two‐tailed Student's *t*‐test using the measurements of the untreated and treated samples as a data set.

It has been shown that PGLYRP4, directly or indirectly, induces CpxA/R TCS (Di Martino, Romilly, et al. 2016; Kashyap et al. 2011). Transcription of the *virF* gene in *Shigella* requires the CpxR regulator to bind to the *virF* promoter after CpxR is phosphorylated by its cognate sensor, CpxA (Pasqua et al. 2022). This regulation was previously well‐characterized in *S. sonnei* (Nakayama and Watanabe 1998; Nakayama and Watanabe 1995), and we have further confirmed it in *S. flexneri* by comparing the transcriptional profile of the *virF* gene in the wild‐type M90T strain and a *cpxA* or *cpxR* defective derivative strain. Results show that while the loss of CpxR almost completely abolishes *virF* transcription, the absence of the CpxA sensor reduces *virF* transcription to 0.3‐fold compared to the wt (Figure S3). To test whether the PGLYRP4 protein‐dependent increase in VirF expression is mediated by CpxA/R TCS activation, we evaluated the transcriptional profile of *virF* by qRT‐PCR in response to the absence or increased concentrations of PGLYRP4 in the M90T Δ*cpxA* strain. This analysis reveals that the transcriptional level of *virF* is comparable at all PGLYRP4 concentrations and does not deviate significantly from the untreated control (Figure 4a). Furthermore, the actual PGLYRP4‐dependent activation of the CpxA/R system and its failure to activate in the CpxA‐deficient mutant was verified by also monitoring the induction of *cpxP* gene (Figure S4), a member of the *cpx* regulon, which is often used as a reporter for CpxA/R activation. In addition, the ineffectiveness of PGLYRP4 in influencing *virF* expression, and therefore the expression of downstream regulated genes, in the absence of CpxA was further confirmed at the translational level. Western blot assays designed to assess the VirF and VirB protein levels in strain M90T Δ*cpxA* treated with increasing concentrations of PGLYRP4 show a comparable amount of both proteins at all concentrations tested (Figure 4b,c). Altogether, these results demonstrate that the CpxA/R TCS mediates the activation of PGLYRP4‐dependent *virF* transcription.

### 3.4 *Shigella* Copes Better With PGLYRP4‐Dependent Oxidative Stress Than *E. coli* and May Attenuate Its Bactericidal Potential

**Figure 5:**
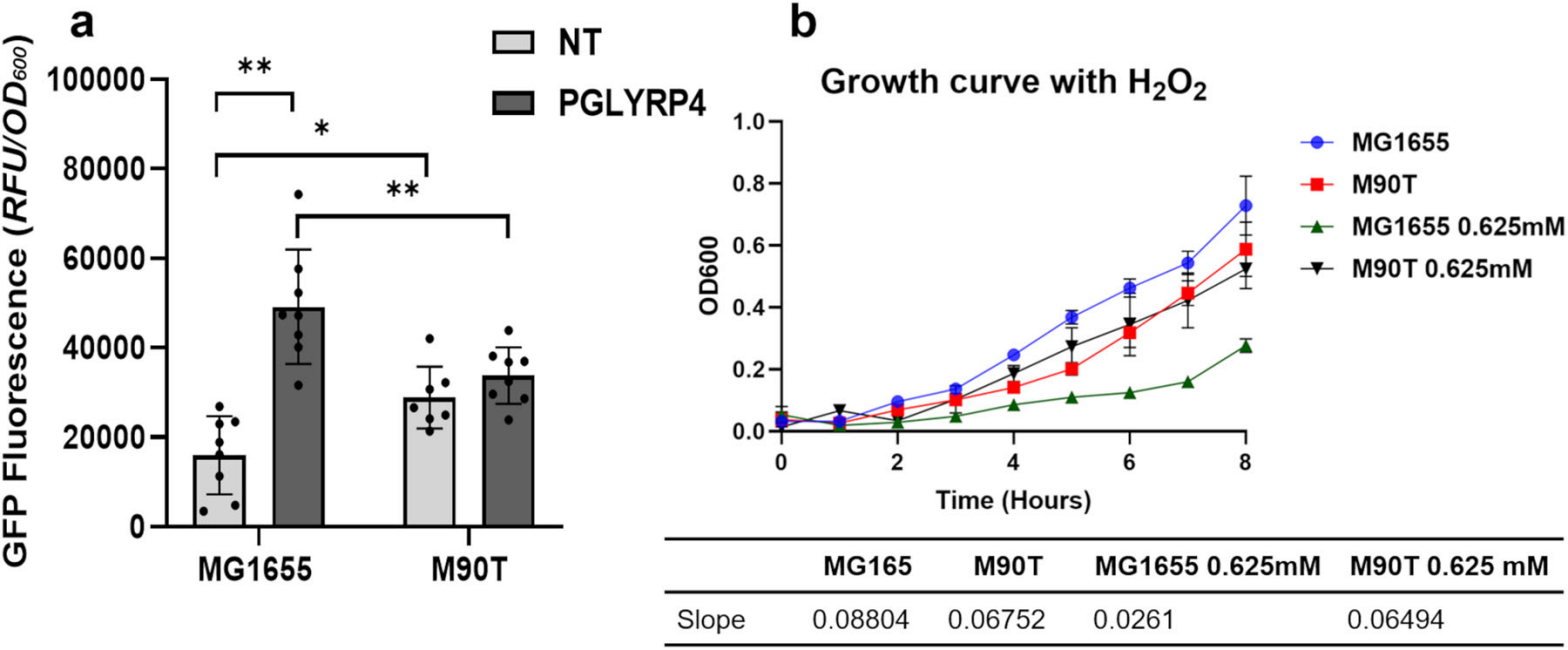
*Shigella* is more responsive to oxidative stress than *E. coli*. (a) GFP levels measured from *E. coli* MG1655 and *S. flexneri* M90T harboring the *ahp*C promoter *gfp* bioreporter (pLA40) grown in LB medium with and without H_2_O_2_. Results are averaged from at least eight independent experiments (*n* = 8) (b) Growth curves of *E. coli* MG1655 and *S. flexneri* M90T with and without H_2_O_2_. The growth rate (slope) is shown below the graph. Results are averaged from at least three independent experiments (*n* = 3). Statistical significance of GFP fluorescence (a) was evaluated by paired two‐tailed Student's *t*‐test (**p ≤ 0.01), while that of growth curves (b) was determined by two‐way analysis of variance (ANOVA) (*p* < 0.0001). Error bars represent SD.

The treatment of bacteria with 100 µg/mL PGLYRP4 induces a combination of thiol, metal, and oxidative stress that, all together, can kill bacteria in vitro and, in association with antimicrobial peptides, also in vivo (Kashyap et al. 2020, 2014; Yang et al. 2021). In Gram‐negative bacteria, the oxidative stress has been correlated with the CpxA/R system, as the PGLYRP4‐induced increase in H_2_O_2_ was absent in the *cpxA* mutant of *E. coli*, whereas thiol and metal stress appeared to be independent of the TCS of CpxA/R (Kashyap et al. 2017). Therefore, given the role of CpxA/R in inducing *virF* transcription in the PGLYRP4‐treated *Shigella* M90T strain, we studied whether *Shigella* exposed to the highest concentration of PGLYRP4 employed in this study also exhibited oxidative stress. To assess the level of oxidative stress caused by the PGLYRP4 protein, we used the pLA40 reporter plasmid, a construct carrying the *ahp*C promoter fused to *gfp* gene (Aussel et al. 2011). The *ahp*C promoter responds to oxidative stress by increasing transcription of the *ahp*C gene, which encodes the alkyl hydroperoxide peroxidase subunit C involved in the reduction of the hydroperoxide substrate (Lu and Holmgren 2014). *E. coli* MG1655 and *S. flexneri* M90T strains, both hosting the pLA40 plasmid, were treated with PGLYRP4, and the fluorescence was measured. The PGLYRP4‐treated *E. coli* strain shows a threefold increase of fluorescence as compared to the untreated control, while *Shigella* exhibits comparable levels in both untreated and treated samples (Figure 5a). It is noteworthy that the fluorescence emitted by the untreated *S. flexneri* sample was significantly higher than observed in the untreated *E. coli* sample (Figure 5a). This difference is not attributable to variations in the copy number of the pLA40 plasmid between *E. coli* and *Shigella* strains, as confirmed by relative qPCR analysis (Figure S5). Moreover, the higher expression of *ahpC* in *Shigella* was validated by qRT‐PCR using the same housekeeping gene for both *E. coli* and *Shigella*. These findings indicate that *Shigella* exhibits a higher basal expression of the *ahpC* and *katG* genes compared to *E. coli* (Figure S6) (Barbagallo et al. 2011). This supports the hypothesis that *Shigella* has evolved a constitutively elevated expression of ROS‐detoxifying genes, likely as an adaptive response to the oxidative stress encountered during infection. To further test this hypothesis, we compared the sensitivity of *Shigella* and *E. coli* to oxidative stress by assessing the growth of the *E. coli* MG1655 and *S. flexneri* M90T strains in the presence of increasing concentrations of H_2_O_2_ (Figure S7). The MIC for *S. flexneri* was twice that of *E. coli* (2.5 mM vs. 1.25 mM H_2_O_2_). The growth curves of the tested bacteria, whether treated with 0.625 mM H_2_O_2_ (half the MIC for *E. coli*) or not, show that the growth of *E. coli* is significantly affected by the H_2_O_2_ treatment, while *Shigella* shows only a slight difference as compared to the untreated control (Figure 5b). Relative specific growth rates are 0.08 and 0.04 for untreated and treated *E. coli* samples, and 0.04 and 0.05 for untreated and treated *Shigella* samples. This confirms that, as compared to non‐pathogenic *E. coli*, *Shigella* has a more ready response to oxidative stress, which probably includes the one induced by PGLYRP4.

## 4 Discussion

In this study, we investigated a novel mechanism by which *Shigella* exploits the PGLYRP4 protein, a component of the host's innate immune system, to increase its virulence at the site of infection without affecting bacterial viability. Furthermore, we showed that *Shigella* evolved a more ready ability to respond to oxidative stress, including that induced by PGLYRP4, thus contributing to coping with the bactericidal effect of this protein.

PGLYRP4 belongs to a group of innate immunity proteins (PGRP) conserved from insects to mammals, and including other invertebrates such as molluscs and echinoderms, and, among vertebrates, fish, amphibians, and birds (Royet and Dziarski 2007; Yoshida et al. 1996). These proteins are characterized by having one or two so‐called PGRP domains, a 165 amino acids domain sharing structural similarity with bacterial and bacteriophage amidase type 2 (Dziarski and Gupta 2006). They consist of three helices surrounding a core of several β‐strands, resulting in a cleft that forms the peptidoglycan‐binding groove (Guan, Malchiodi, et al. 2004), which interacts with muramyl pentapeptides or tetrapeptides with an affinity degree that depends on the amino acid residues (Guan, Roychowdhury, et al. 2004). Some PGRP domains show amidase activity, while others have lost this ability while still retaining binding affinity for PG fragments (Dziarski 2004; Kang et al. 1998; Werner et al. 2000). PGRP proteins often display a hydrophobic domain that is believed to be involved in binding with other microbial molecules, such as lipopolysaccharide (LPS), lipoteichoic acid, and fungus‐derived molecules (M.‐S. Kim et al. 2003; Liu et al. 2000; Sharma et al. 2011; Tydell et al. 2006).

**Figure 6:**
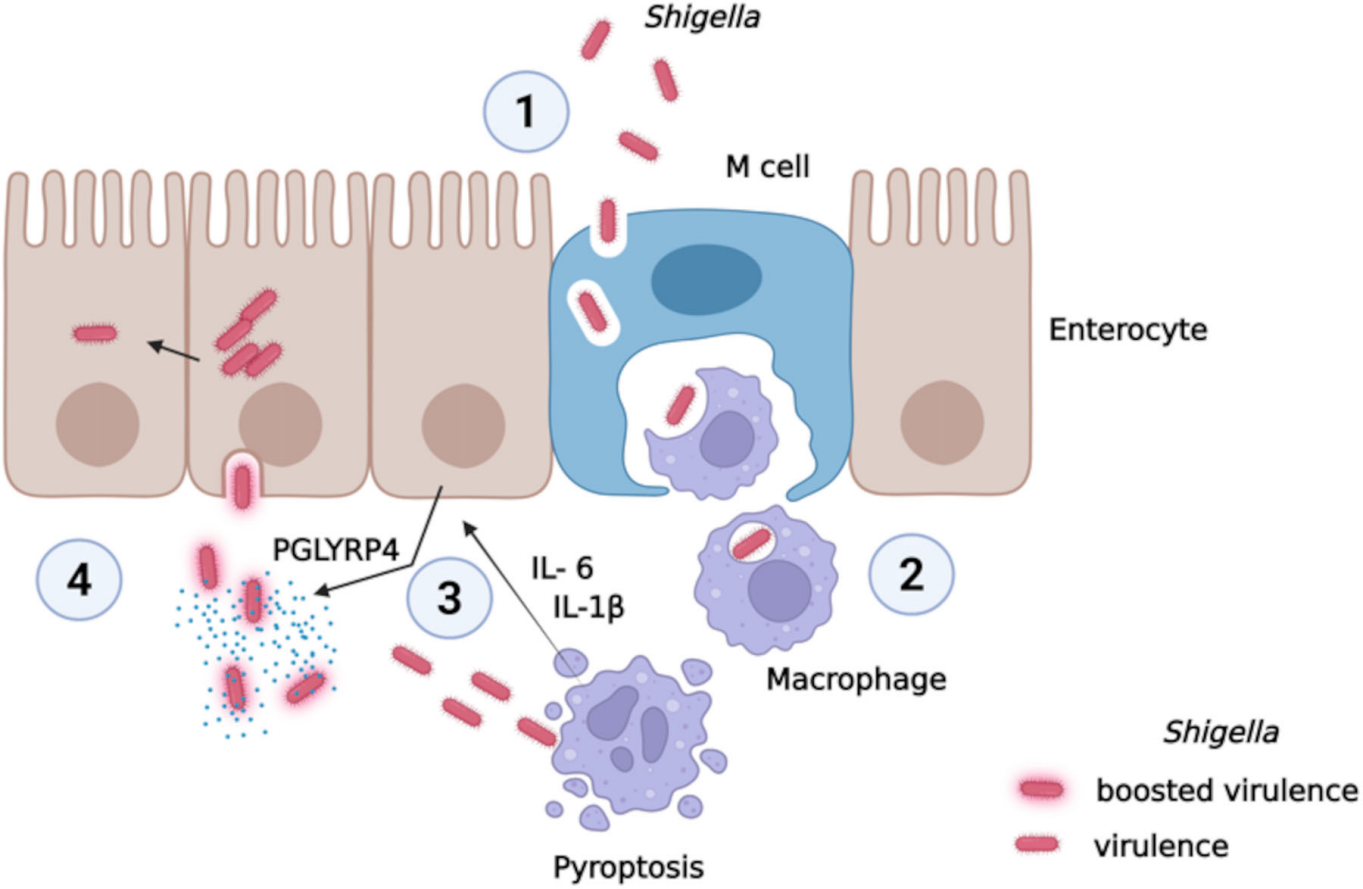
Proposed model of the role of PGLYRP4 in *Shigella* invasion. *Shigella* crosses the intestinal barrier by exploiting the transcytotic properties of M cells. 2. Once *Shigella* is released into the intraepithelial pocket, resident macrophages intercept and phagocytose it. 3. *Shigella* induces pyroptosis, and dying macrophages release pro‐inflammatory cytokines, typically IL‐1β and IL‐6, which induce the expression and release of PGLYRP4 by enterocytes. 4. PGLYRP4 enhances *Shigella* virulence to promote enterocyte invasion (Biorender content ‐ PR28CQT5YW).

Mammals, including humans, have four PGRPs, called PGLYRP1, 2, 3, and 4. PGLYRP1 and 2 have a single PGRP domain, while PGLYRP3 and 4 have two non‐identical and homologous PGRP domains, which share the same structural organization. As opposed to the PGRPs in insects, which are membrane proteins working as receptors, mammalian PGLYRPs are secreted as disulfide‐linked homo and heterodimers (Royet et al. 2011). PGLYRP2, which conserves amidase activity and acts as a monomer, is constitutively expressed in the liver and secreted into the bloodstream (Royet and Dziarski 2007; Z.‐M. Wang et al. 2003), and is induced by cytokines in epithelial cells (Uehara et al. 2005; H. Wang et al. 2005). PGLYRP1 is found mainly in activated neutrophil and eosinophil granules (Cho et al. 2005). PGLYRP3 and PGLYRP4 are expressed in the skin, eyes, oral cavity, and gastrointestinal tract, where they may be involved in maintaining a healthy gut microbiome by promoting the maintenance of beneficial gut microbiota (Royet et al. 2011; Saha et al. 2010). To date, the tissue‐specific expression of PGLYRP proteins has been only loosely mapped, and data on their actual concentration in human tissues remain scarce. Concerning the lumen and tissues of the human colon, the currently available data are sometimes contradictory and do not allow for an estimation of physiological PGLYRP protein concentrations; therefore, this remains uncertain. In fact, a human tissue proteome database, based on an integrated omics approach, does not report the presence of PGLYRP proteins in the human colon, including PGLYRP4 (Uhlén et al. 2015), while other experimental data show that PGLYRP4 and PGLYRP3 are expressed in apical colon‐absorbing epithelial cells of the healthy human small intestine and, at significantly lower levels, in the colon (Lu et al. 2006). Based on these experimental data, we believe it is reasonable to hypothesize that in the healthy human colon, the PGLYRP4 protein could be expressed at a low level, probably far from bactericidal concentration, whereas its expression probably increases under pathological conditions following induction dependent on pro‐inflammatory cytokines (Chen et al. 2025). These observations allow some speculations about the specific stage of the *Shigella* infection at which PGLYRP4 may act to influence the expression of virulence. One hypothesis is based on the possible role of PGLYRP proteins secreted in the human gut in maintaining a beneficial microbial community. While PGLYRP4 is involved in this effect, it may also contribute, together with other environmental stimuli (pH, temperature, osmolarity, etc.), to support the expression of the virulence system of *Shigella* (Marteyn et al. 2012). A more intriguing hypothesis stems from the fact that dying macrophages, which phagocytize *Shigella* once it crosses the intestinal mucosa and in which *Shigella* induces pyroptosis, release pro‐inflammatory cytokines. Among these, IL‐1β and IL‐6 (Shabani et al. 2020) trigger various inflammatory responses, including the induction and release of PGLYRP4 by enterocytes (Chen et al. 2025). In this scenario, we can hypothesize that, when *Shigella* escapes from dying macrophages in the lamina propria, the resulting increase in local PGLYRP4 concentration could enhance *Shigella* virulence system. This would improve the expression of virulence factors and ensure the invasion of enterocytes (Figure 6). Both hypotheses are well supported by the results obtained by transcriptional and translational analyses in this study, which show a dose‐dependent induction of *virF* expression by PGLYRP4 (Figure 1a,b), as opposed to PGLYRP1, 2, and 3, resulting in an enhanced ability of *Shigella* to invade Caco‐2 epithelial cells in vitro (Figure 2a,b). In addition, by comparing the response to PGLYRP4‐induced oxidative stress of *Shigella* and a commensal *E. coli*, we showed that the former is better able to withstand oxidative stress than the latter (Figure 5a). The results of the growth curve analysis of H_2_O_2_‐treated *Shigella* and *E. coli* cultures further support this assumption (Figure 5b). These results suggest that *Shigella*'s ROS detoxification system could prevent the induction of PGLYRP4‐mediated bacterial killing by inactivating an essential stress that, if simultaneous with the thiol and metal stress, contributes to the killing effect (Kashyap et al. 2014, 2017).

Interestingly, in our experiments, PGLYRP3 does not show comparable effects to PGLYRP4 regarding *Shigella* virulence system induction, infectivity, and oxidative stress. Although these two proteins share a homologous structure, almost the same expression pattern, similar biological functions, and can form heterodimers (Lu et al. 2006), they differ in expression level, molecular weight, glycosylation pattern, and immunoreactivity. They also show different spectra of bactericidal activity against specific bacteria and diverse efficacies in protecting *S. aureus*‐infected mice from lung infection (Lu et al. 2006). These differences suggest that the two proteins could also have distinct roles. Moreover, although PGLYRP3 and 4 share a 67% amino acid sequence identity, PGLYRP4 has an extra 34 amino acid stretch, from position 22 to 55 (Figure 8S), which is predicted to form an alpha‐helix adjacent to the amino‐terminal sequence containing the hydrophobic domain (position 1–17) (Bateman et al. 2025) that has been hypothesized to interact with the LPS of Gram‐negative bacteria (Royet and Dziarski 2007). The presence of this additional sequence may contribute to the diverse effects of PGLYRP4 and 3 on *Shigella* virulence.

Overall, our findings clearly demonstrate that PGLYRP4 stimulates the expression of *virF*, the master regulator of the *Shigella* virulence system, strongly supporting our hypotheses. However, further studies are needed to determine the physiological concentration of PGLYRP4 in human colonic tissue and to assess its secretion by epithelial cells following cytokine stimulation. Moreover, in vivo experiments will be crucial to validate these findings. Additionally, increasing the concentrations of PGLYRP4, as well as other PGLYRPs, in both in vitro and ex vivo experiments could deepen our understanding of their role in modulating *Shigella* virulence.

More broadly, the bactericidal and bacteriostatic activity of PGLYRP1, 3, and 4 against both Gram‐positive and Gram‐negative bacteria has been shown to induce the activation of the CssS/R and CpxA/R TCSs, respectively (Kashyap et al. 2011, 2014). Notably, the CpxA/R system also regulates virulence gene expression in several Gram‐negative pathogens beyond *Shigella* (Pasqua et al. 2022; Raivio 2014). These observations suggest that other Gram‐negative bacteria may have evolved mechanisms to sense and exploit host‐derived PGLYRPs to modulate their own virulence programs within immunoreactive tissues. Thus, this strategy may represent a broader adaptive mechanism not limited to *Shigella*, but potentially common among diverse bacterial pathogens.

## 5 Conclusions

Our work presents evidence for a yet unknown role of PGLYRP4 in locus‐specific modulation of the expression of virulence in *Shigella*. A modulation that is a highly effective adaptation of *Shigella* to the host environment, as it considers the detection of a specific level of host immune activation through the perception of immune factors that cause adaptive variation in pathogen behavior. PGLYRP4 may thus be added to the list of specific signals of the host inflammatory response, such as cytokines and immune mediators, previously described as being involved in the adaptation of pathogenic bacteria that evolve into the microbial ability to sense and respond to host inflammatory activation (Hitzler et al. 2025). A comprehensive understanding of the molecular processes underlying these adaptive pathogenic strategies could lead to the discovery of potential targets for diagnostic and therapeutic approaches, thereby improving strategies to prevent and control bacterial virulence and pathogenicity.

## Author Contributions

**Rita Trirocco** and **Gianni Prosseda:** conceptualization and design of the study, analysis, and interpretation of data. **Rita Trirocco:** visualization, methodology, investigation. **Gianni Prosseda:** writing – original draft, funding acquisition. **Rita Trirocco** and **Gianni Prosseda:** review and editing.

## Ethics Statement

The authors have nothing to report.

## Conflicts of Interest

The authors declare no conflicts of interest.

## Supporting information

Supplementary.

## Data Availability

Data will be made available on request.
