# Supplementary material for: PGLYRP4 Enhances Shigella flexneri Virulence by Promoting virF Transcription via the CpxA/R Two‐Component System
Source: Microbiologyopen. 2025 Nov 9;14(6):e70156. doi: 10.1002/mbo3.70156 (PMC12597783; doi:10.1002/mbo3.70156)

**Supplementary: Tables**

**Table 1S: Strains and plasmids used in this study**


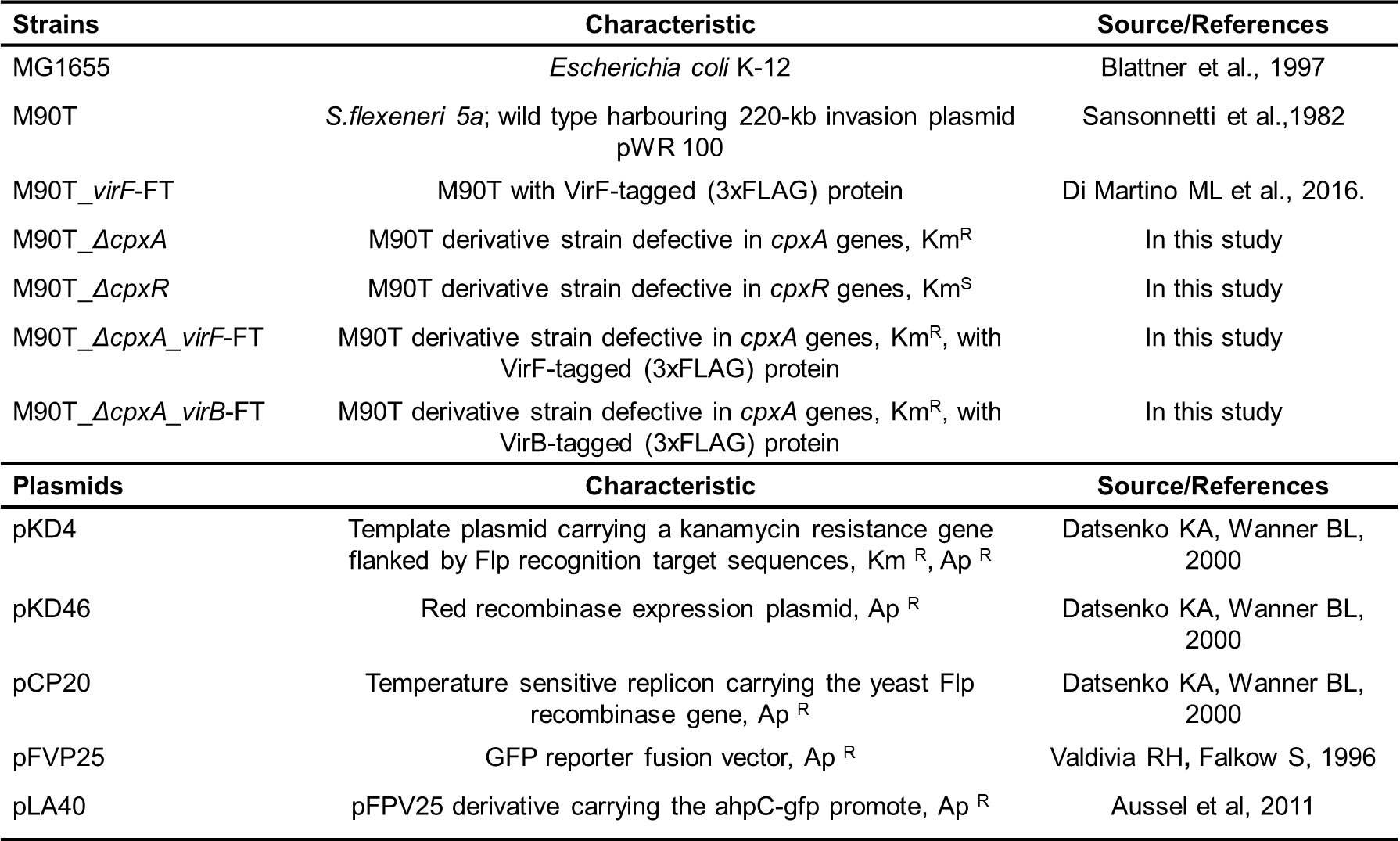


**Table 2S: Oligos used in this study**


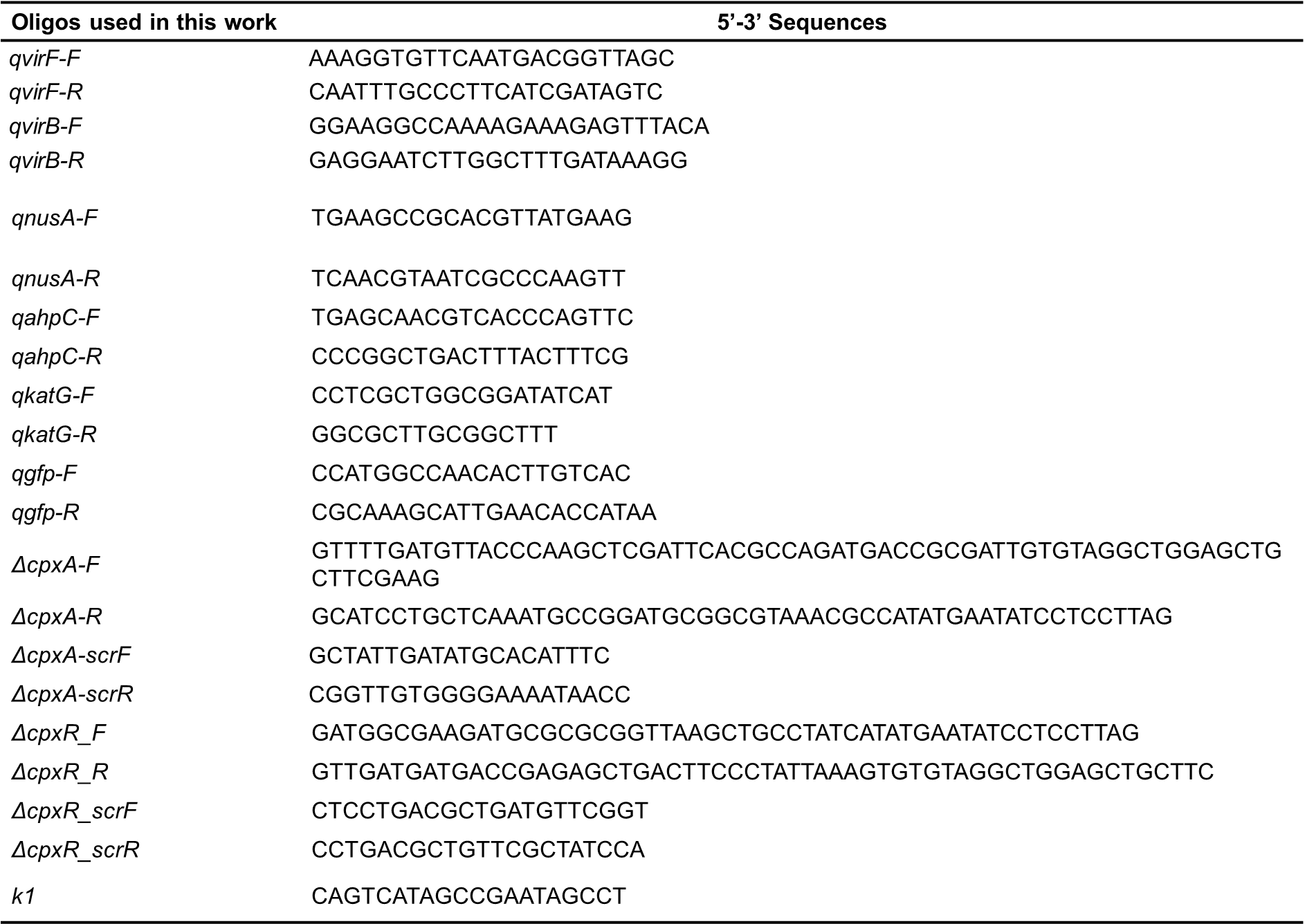


**Bibliography of Strain and Plasmid Tables.**

Aussel, L., Zhao, W., Hébrard, M., Guilhon, A. A., Viala, J. P., Henri, S., Chasson, L., Gorvel, J. P., Barras, F., & Méresse, S. (2011). *Salmonella* detoxifying enzymes are sufficient to cope with the host oxidative burst. Molecular microbiology, 80(3), 628–640. <https://doi.org/10.1111/j.1365-2958.2011.07611.x>

Blattner, F. R., Plunkett, G., 3rd, Bloch, C. A., Perna, N. T., Burland, V., Riley, M., Collado-Vides, J., Glasner, J. D., Rode, C. K., Mayhew, G. F., Gregor, J., Davis, N. W., Kirkpatrick, H. A., Goeden, M. A., Rose, D. J., Mau, B., & Shao, Y. (1997). The complete genome sequence of *Escherichia coli* K-12. Science (New York, N.Y.), 277(5331), 1453–1462. <https://doi.org/10.1126/science.277.5331.1453>

Datsenko, K. A., & Wanner, B. L. (2000). One-step inactivation of chromosomal genes in *Escherichia coli* K-12 using PCR products. Proceedings of the National Academy of Sciences of the United States of America, 97(12), 6640–6645. <https://doi.org/10.1073/pnas.120163297>

Di Martino, M. L., Falconi, M., Micheli, G., Colonna, B., & Prosseda, G. (2016). The Multifaceted Activity of the VirF Regulatory Protein in the *Shigella* Lifestyle. Frontiers in molecular biosciences, 3, 61. <https://doi.org/10.3389/fmolb.2016.00061>

Sansonetti, P. J., Kopecko, D. J., & Formal, S. B. (1982). Involvement of a plasmid in the invasive ability of *Shigella flexneri*. Infection and immunity, 35(3), 852–860. <https://doi.org/10.1128/iai.35.3.852-860.1982>

Valdivia, R. H., & Falkow, S. (1996). Bacterial genetics by flow cytometry: rapid isolation of Salmonella typhimurium acid-inducible promoters by differential fluorescence induction. Molecular microbiology, 22(2), 367–378. https://doi.org/10.1046/j.1365-2958.1996.00120.x

**Supplementary: Figures**

**Figure 1S: *S. flexneri* M90T growth curves in the presence of increasing (0.05 to 2 µg/mL) concentrations of PGLYRP proteins (1-4). (error bars indicate SD, n=3)**

**
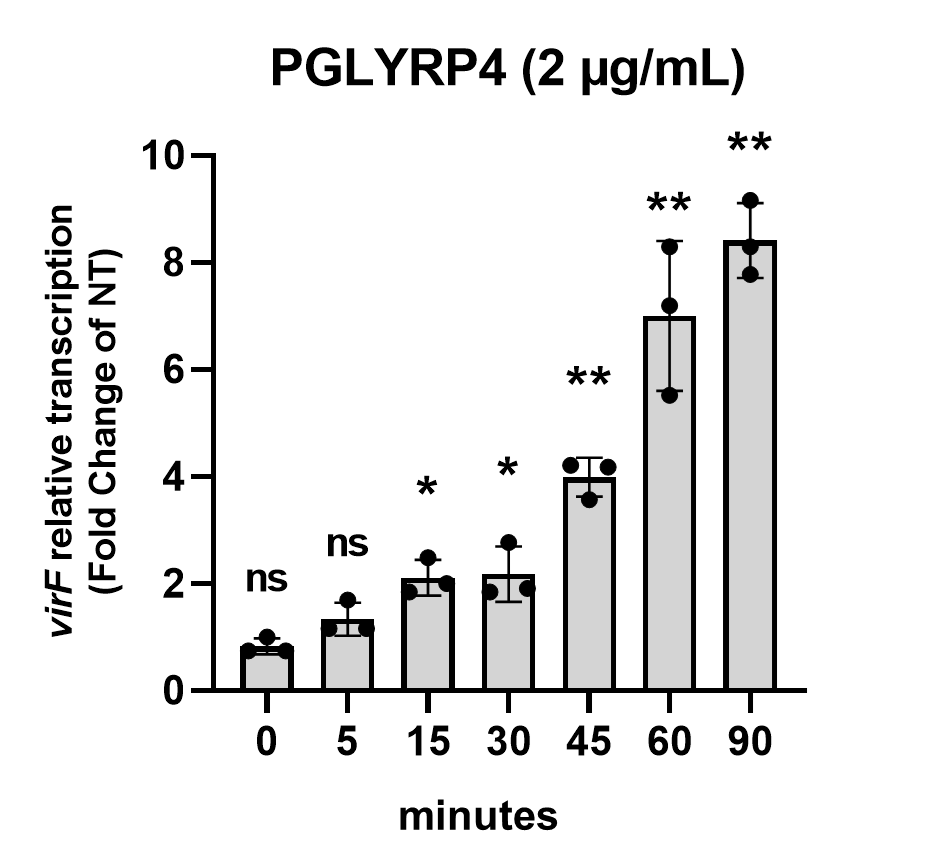
Figure 2S: PGLYRP4 time-dependent induction of *virF* transcription (n=3, error bars indicate SD, * p value <0.05, ** p value <0.01).**

**Figure 3S: Transcriptional profile of the *virF* gene in *S. flexneri* M90T wt, *cpxA* or *cpxR* defective strains (error bars indicate SD, * p value <0.05).**

**Figure 4S: *cpxP* PGLYRP4-dependent induction (n= 3, error bars indicate SD, * p value <0.05, ** p value <0.01).**

**
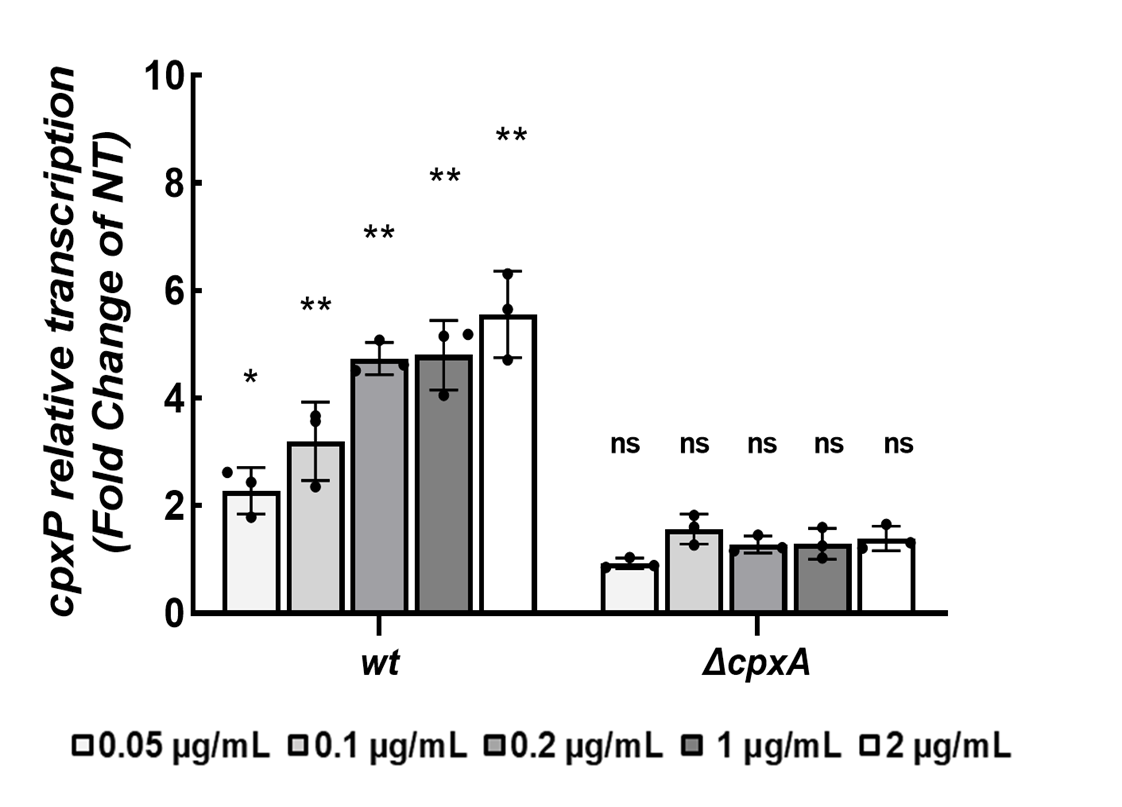
**

**Figure 5S: relative abundance of *gfp* plasmid gene relative to the *nusA* chromosomal gene (n=6, error bars indicate SD).**

**Figure 6S: comparison of *ahpC* and *katG* gene relative to the expression of *nusA* gene used as reference in qRT-PCR experiment** **(n=3, error bars indicate SD, ** p value <0.001.**

**Figure 7S: Hydrogen peroxide Minimum Inhibitory Concentration (MIC) for *E. coli* MG1655 (a) and *S. flexneri* M90T strain (b). Both strains were treated with increasing concentrations of H_2_O_2,_ and bacterial growth was detected at the time of inoculum (T0) and after 8 hours (T8). The red arrow indicates the MIC value (n=3, error bars indicate SD, ** p value <0.01).**

b

a


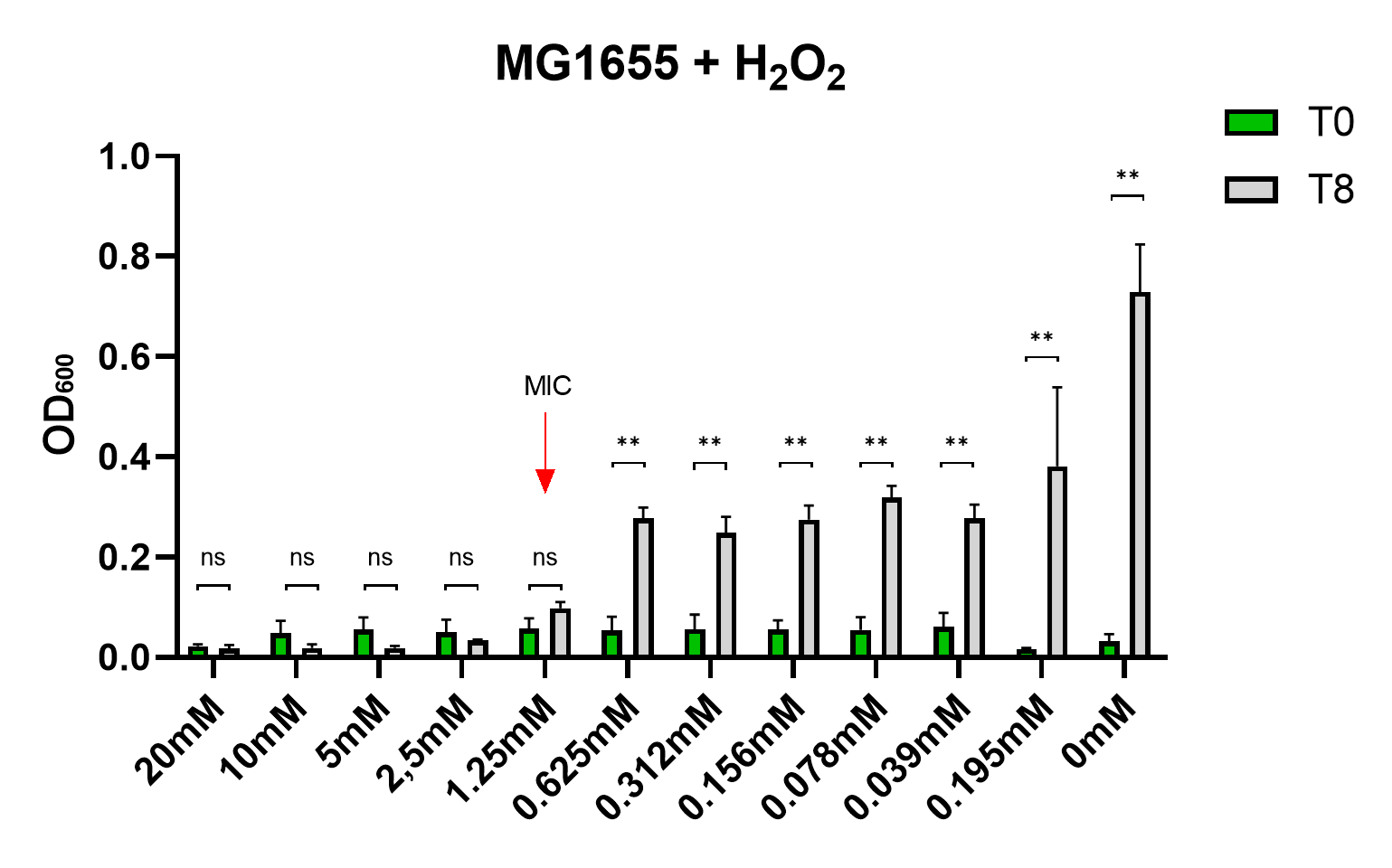


**Figure 8S: PGLYRP4 (Q96LB9 · PGRP3_HUMAN) and PGLYRP3 (Q96LB9 · PGRP3_HUMAN) sequence alignment result. The sequence boxed in red indicates the additional 34 amino acid stretch present in PGLYRP4 and absent in PGLYRP3 (**[**https://www.uniprot.org/align**](https://www.uniprot.org/align)**).**

**
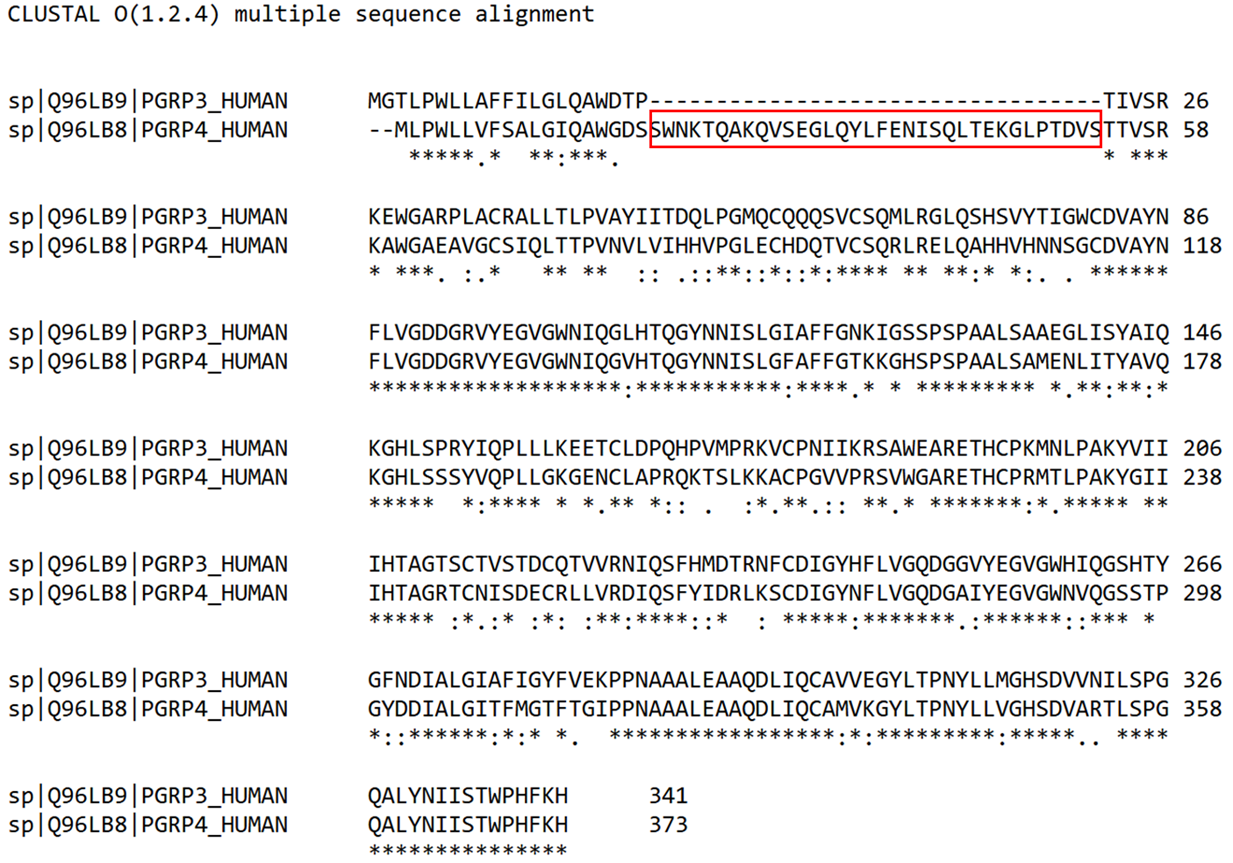
**

**Fig. 9S: Original western blot images used for Western Blot figure.**


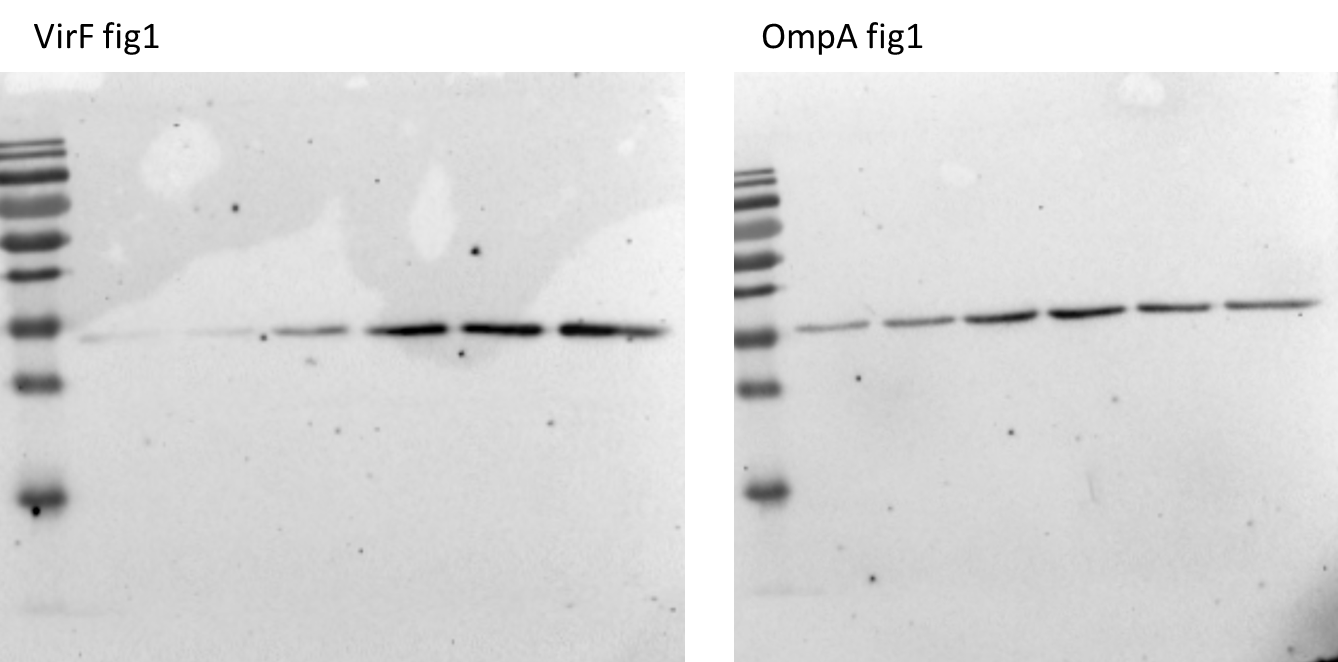


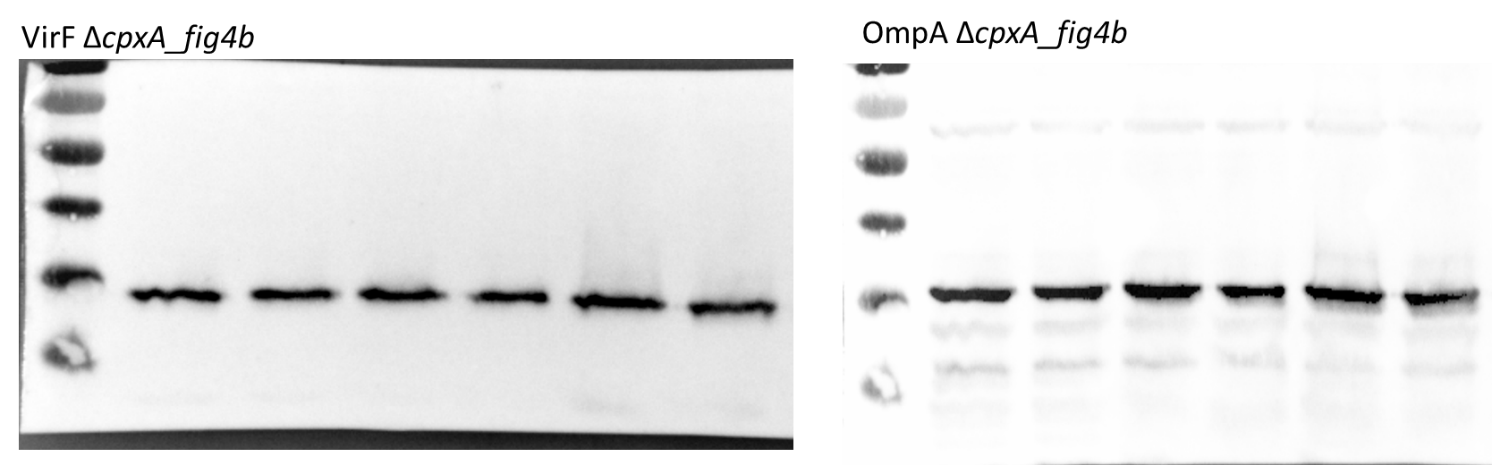


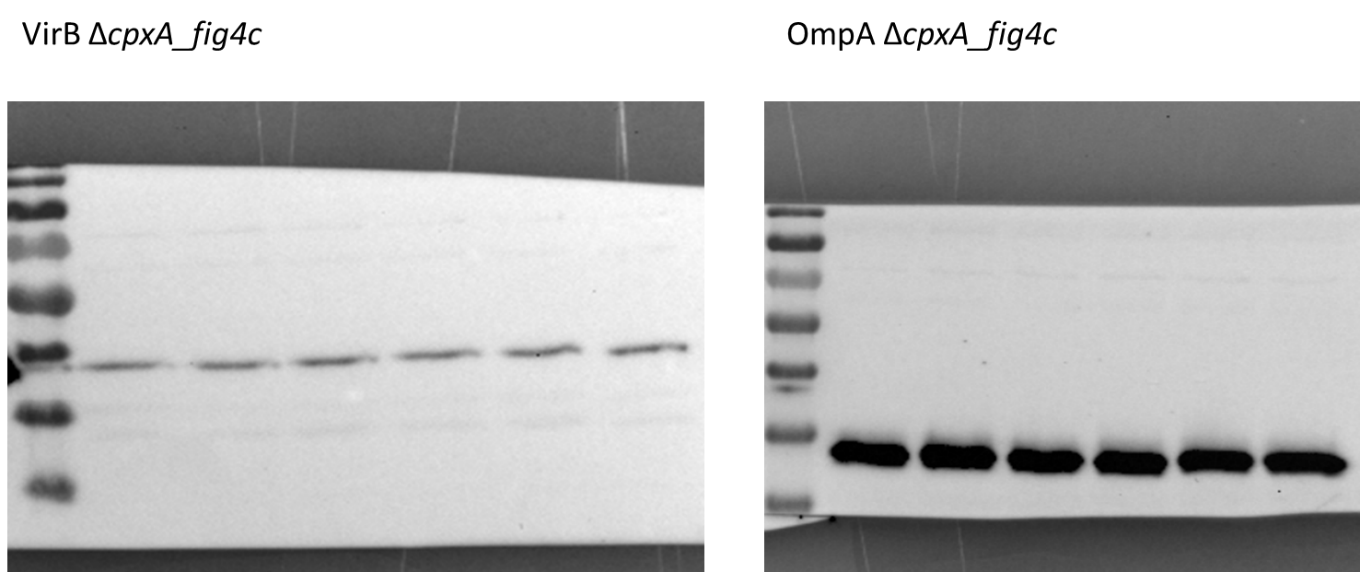

Supplement: Supplementary file 1 — Supplementary. [file MBO3-14-e70156-s001.docx]
